# Holographic Fabrication of 3D Moiré Photonic Crystals Using Circularly Polarized Laser Beams and a Spatial Light Modulator

**DOI:** 10.3390/mi14061217

**Published:** 2023-06-09

**Authors:** Noah Hurley, Steve Kamau, Jingbiao Cui, Yuankun Lin

**Affiliations:** 1 Department of Physics, University of North Texas, Denton, TX 76203, USA; noahhurley@my.unt.edu (N.H.); stevekamau@my.unt.edu (S.K.); jingbiao.cui@unt.edu (J.C.); 2 Department of Electrical Engineering, University of North Texas, Denton, TX 76203, USA

**Keywords:** micro-/nanofabrication, holographic lithography, interference lithography, laser fabrication, micro-/nanostructures, photonic crystal, spatial light modulator-based lithography

## Abstract

A moiré photonic crystal is an optical analog of twisted graphene. A 3D moiré photonic crystal is a new nano-/microstructure that is distinguished from bilayer twisted photonic crystals. Holographic fabrication of a 3D moiré photonic crystal is very difficult due to the coexistence of the bright and dark regions, where the exposure threshold is suitable for one region but not for the other. In this paper, we study the holographic fabrication of 3D moiré photonic crystals using an integrated system of a single reflective optical element (ROE) and a spatial light modulator (SLM) where nine beams (four inner beams + four outer beams + central beam) are overlapped. By modifying the phase and amplitude of the interfering beams, the interference patterns of 3D moiré photonic crystals are systemically simulated and compared with the holographic structures to gain a comprehensive understanding of SLM-based holographic fabrication. We report the holographic fabrication of phase and beam intensity ratio-dependent 3D moiré photonic crystals and their structural characterization. Superlattices modulated in the z-direction of 3D moiré photonic crystals have been discovered. This comprehensive study provides guidance for future pixel-by-pixel phase engineering in SLM for complex holographic structures.

## 1. Introduction

Three-dimensional (3D) fabrication can be realized by either top-down or bottom-up approaches. The bottom-up approach aims to build complex structures through self-assembly [1] or additive manufacturing [2]. The top-down approach, on the other hand, relies on the selective removal of bulk materials using methods such as deep UV [3], focused ion beam [4,5], or e-beam lithography [6]. In particular, 3D photonic crystals, which are a periodic array of binary dielectric materials with a photonic bandgap, have been produced using top-down layer-by-layer lithography [7], bottom-up nano-sphere self-assembly [8], or direct laser writing with small feature sizes and flexible lattice symmetry [9]. Another prominent 3D nanofabrication approach for 3D photonic crystals is through multi-beam laser holographic lithography [10,11,12], which is considered one of the most attractive approaches for its controllability, flexibility, and most importantly, scalability due to its capability for the parallel and simultaneous fabrication of large area structures in one exposure.

A liquid crystal-based spatial light modulator (SLM) is a dynamic and reconfigurable optical element that allows the real-time control of phase patterns [2,13,14,15]. These phase patterns displayed in the SLM can modulate the phase and amplitude of incident light with exceptional spatial precision [2,13,14,15]. SLM-based holographic lithography has significantly reduced optical setup complexity and demonstrated capability to fabricate two-dimensional (2D) photonic crystals with desired defects [16,17,18,19], quasi-photonic crystals [20,21], graded photonic crystals [22,23,24,25], and moiré photonic crystals with unit super-cells in square [26,27,28], rectangular [29], and triangular [26] symmetries, together with other methods [30,31,32].

The moiré effect is an old phenomenon that creates a moiré pattern that is usually formed when two repetitive structures are superimposed against each other with a relative rotation angle (twist angle) [33]. Recently, twisted bilayers of two-dimensional (2D) materials have ignited significant interest due to the ability of experiments to control the relative twist angle between successive layers [34,35,36]. Manipulating the twist angle between the two layers of 2D materials has resulted in a fine control of the electronic band structure [37], magic-angle flat-band superconductivity [38], the formation of moiré excitons [39], interlayer magnetism [40,41], topological edge states [42], and correlated insulator phases [43]. Twisted moiré photonic crystal is an optical analog of twisted 2D materials, with significant interest triggered by the discovery of flat photonic bands in moiré photonic crystal [44,45,46,47,48,49]. A 3D moiré photonic crystal is a new nano-/microstructure that distinguishes itself from the bilayer twisted photonic crystals. The research on 3D moiré photonic crystals is still in its infancy. We initially fabricated 3D moiré photonic crystals through SLM-based holographic lithography using nine-beam interference [50]. Due to the complex unit super-cells that have been used in the phase pattern, a comprehensive understanding of the relationship between the gray level setting in the SLM and the generated holographic pattern was missing [50]. Furthermore, many intriguing structures that surpass our imagination have yet to be explored.

In this paper, we follow two paths to understand the relationship between the pixel-by-pixel gray level setting and the 3D holographic pattern through simulation and structural characterization: (1) we study the 3D moiré photonic crystal dependence on the inner/outer beam intensity ratio while the phase shift among the outer beam is the same; (2) we study the 3D moiré photonic crystal dependence on the phase shift among inner beams while the inner/outer beam intensity ratio is the same. Three-dimensional moiré photonic crystals are fabricated, explained by the simulation, and characterized by diffraction and scanning electron microscopy (SEM). The super-lattices modulated in the z-direction of 3D moiré photonic crystals are discovered.

## 2. Materials and Methods

All 3D moiré photonic crystals were fabricated using a photosensitive chemical mixture named dipentaerythritol hexa/pentaacrylate (DPHPA). The mixture is a modified DPHPA monomer with a mass percentage of 88.62% m/m in the final solution. Three chemicals are added to improve the sensitivity of the mixture to 532 nm light and are as follows: rose-bengal photo-initiator (RB), co-initiator N-phenylglycine (NPG), and chain extender N-vinylpyrrolidone (NVP), with mass percentages of 0.1% m/m, 0.80% m/m, and 10.48% m/m, respectively. DPHPA is not an effective solvent and must follow a mixing procedure to ensure a smooth solution. First, mix the RB, NVP, and NPG in an amber bottle and sonicate in a water bath for thirty minutes. After sonication, stir the solution with a magnetic stir-bar for approximately one hour at room temperature. While the three chemicals are stirring, measure out the DPHPA monomer in a separate bottle. Heat a beaker full of water to 90 °C and place the DPHPA bottle in the heated water. Allow the DPHPA to heat until its viscosity resembles that of water and it flows easily. Pour the heated DPHPA into the bottle containing RB, NVP, and NPG, then stir via a magnetic stir-bar overnight without any additional heat and at room temperature. Note that spin-coating can be used to gauge the quality of the DPHPA mixture. If the coated glass slide has a smooth surface with few particulates, the solution is well mixed; if the solution is grainy with many tiny particulates on the surface, the solution is poorly mixed, which results in messy holographic structures that have large breaks. The reliability of this process is very good when the sonication time is optimized and the DPHPA is heated to a watery viscosity to allow good mixing and dissolving of the solid components of the mixture. The yield of this process is all or nothing, with the mixture being either very good or very poor. This modified DPHPA mixture was then spin-coated onto a slide and exposed to 532 nm light. After exposure, the samples were developed in 1-methoxy-2-propanol acetate for one hour, rinsed in isopropyl alcohol for one minute, and allowed to air dry.

**Figure 1:**
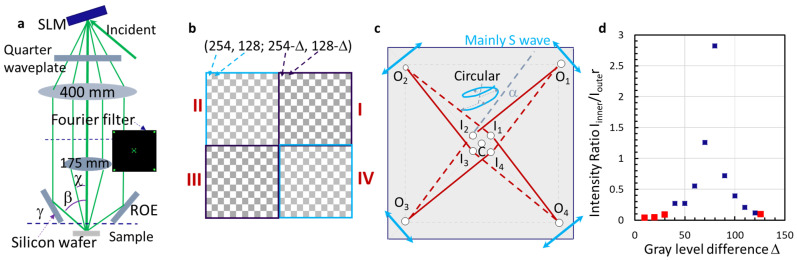
(**a**) Schematic of the experimental setup where the incident light beam is phase-modulated by SLM and becomes circularly polarized after the quarter waveplate. (**b**) A super-cell unit of phase patterns in SLM is divided into 4 quadrants, and gray levels of 254, 128 are arranged in checkerboard format in quadrants II and IV, and gray levels of 254-∆, 128-∆ are arranged in checkerboard format in quadrants I and III. (**c**) Schematic of the nine-interfering beam arrangement: one central and four inner beams are circularly polarized, and four outer beams are s-waves. (**d**) The intensity ratio of the inner beam over the outer beam as a function of gray level is different between quadrants (II, IV) and (I, III) in (**b**). These data points are highlighted in red for the gray level and intensity ratio used in experiments.

Figure 1a shows a schematic of the experimental setup. The designed phase pattern is displayed by the SLM, and the incident light beam (532 nm) is phase-modulated by the phase pattern of the SLM. The diffracted beams of the SLM pass through a quarter waveplate and become circularly polarized. A Fourier filter selects the central beam, four inner beams, and four outer beams, as shown in the figure. These four inner beams and central beams are imaged through the 4f imaging system (f_1_ = 400 mm and f_2_ = 175 mm), and the outer beams are reflected via four polished silicon wafers on the single reflective optical element (ROE) that was made via a 3D printer. An example of super-unit phase patterns in the SLM is shown in Figure 1b, where gray levels of (254, 128) are arranged in a checkerboard pattern in quadrants II and IV, and gray levels of (254-∆, 128-∆) are arranged in a checkerboard pattern in quadrants I and III. The pixeled phase shift of the outer beams diffracted from quadrants II and IV is determined by the gray level difference of (254, 128), while quadrants I and III are determined by the difference (254-∆, 128-∆) [25,51]. Due to the above reason, the phase shift of the outer beams is the same in the design of the phase pattern in Figure 1b [25,51]. The incident angle onto the silicon wafer surface equals the tilt angle, which is 83 deg [27]. With the Brewster angle of the silicon surface being 76.5 deg at 532 nm [52], 90% of the reflected beams (O_1_, O_2_, O_3_, and O_4_, as shown in Figure 1c) are s-waves. The circles in Figure 1c indicate the position of the outer beams (O_1_, O_2_, O_3_, and O_4_), inner beams (I_1_, I_2_, I_3_, and I_4_), and the central beam (C) after passing through the Fourier filter. The intensity of the inner beams and outer beams was measured, and the intensity ratio of the inner beam over the outer beam is plotted in Figure 1d as a function of the gray level difference D, as defined in Figure 1b. At a gray level difference of 10, the intensity ratio is 0.03 and increases to 2.8 when ∆ = 80.

Although the design phase pattern is complex, it utilizes the highest pixel resolution in SLM. This allows for phase and amplitude control of diffracted beams through gray-level settings. Most importantly, holographic fabrication can be performed for phase patterns with various super-cell sizes in SLM without requiring changes to the optical setup depicted in Figure 1a. This makes it compatible with additive manufacturing.

## 3. Theoretical Description and Simulation

**Figure 2:**
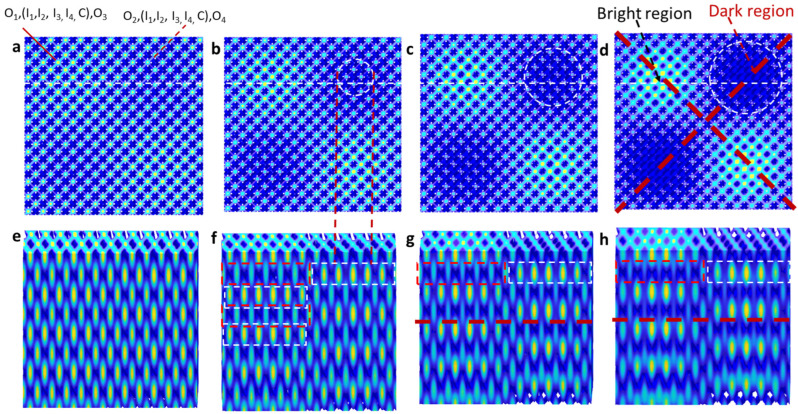
(**a**–**d**) Simulated x–y plane interference patterns with a ratio of inner beam intensity over outer beam intensity of 0.005, 0.03, 0.08, and 0.25, respectively, and (**e**–**h**) their x–z plane interference patterns that were cut along the dashed white line in (**a**–**d**).

All nine beams (O_1_, O_2_, O_3_, O_4_, I_1_, I_2_, I_3_, and I_4_, C) in Figure 1c are overlapped at the sample location as indicated in Figure 1a. The intensity of the nine-beam interference, *I(r)*, is determined by Equation (1):

(1) $$I\left(r\right)=\langle \sum_{i=1}^{9}E_{i}^{2}\left(r,t\right)\rangle +\sum_{i<j}^{9}E_{i}E_{j}{\hat{e}}_{i}\cdot {\hat{e}}_{j}cos\left[\left(k_{j}-k_{i}\right)\cdot r+(\delta_{j}-\delta_{i})\right]$$

where *e*, *E*, *k*, and *δ* are the electric field polarization direction and amplitude, the wave vector, and the initial phase, respectively. The interference pattern is simulated in Matlab (version R2021b) in Figure 2.

In a simplified model, the s-waves of (O_1_, O_3_) and (O_2_, O_4_) are perpendicular in polarization and thus will not interfere with each other. An interference of (O_1_, (I_1_, I_2_, I_3_, I_4_, C), O_3_) forms a pattern in an orientation indicated by a red solid line in Figure 2a, while the interference of (O_2_, (I_1_, I_2_, I_3_, I_4_, C), O_4_) is indicated by the red dashed line. Overall, the interference orientation and period are determined by the wave-vector difference k_n_ − k_m_. The square pattern formed by the wave-vector difference k_n_ − k_m_ indicated by four solid lines in Figure 1c is twisted by an angle of α, away from the pattern formed by k_n_ − k_m_ indicated by four dashed lines [28,53].

Figure 2a–d show simulated x–y plane interference patterns of nine beams with intensity ratios I_inner_/I_outer_ of 0.005, 0.03, 0.08, and 0.25, respectively. We should point out that the simulation was performed for a super unit-cell size of 12a × 12a (where a is the period of a traditional photonic crystal) in order to have a high-resolution pattern in a reasonable time. In the case of I_inner_/I_outer_ = 0.005, the difference between bright and dark zones is barely visible. With an increasing intensity ratio, the dark region becomes more visible. Figure 2e–h shows x–z plane interference patterns that were cut through the 3D interference pattern at the y location with a quarter of the side length from the top, indicated by the dashed white line in Figure 2a–d, respectively. For the case of I_inner_/I_outer_ = 0.005, the interference pattern in the x–z plane is almost uniform in Figure 2e. In Figure 2f–h, the interference in the x–z plane is separated into two regions: the left region with alternating high, low, high, low intensity patterns, and the right region with low, high, low, high intensity patterns from the top to the bottom. These low- and high-intensity patterns are indicated by red and white rectangles in Figure 2f, respectively. In Figure 2h, the intensity difference between the low and high parts is very large; it imposes difficulty in the selection of exposure conditions to obtain a well-developed sample. Through our simulations, we predict that the I_inner_/I_outer_ ratio should be less than 0.3 to ensure the survival and good development of the holographic structure. This is the reason why we selected four specific gray level settings and intensity ratios, highlighted in red in Figure 1d, for the holographic fabrication in the next section. For a gray level difference of 30, our simulation predicts an exposure threshold ranging between 10% and 30% of the maximum intensity.

## 4. Holographic Fabrication Results

**Figure 3:**
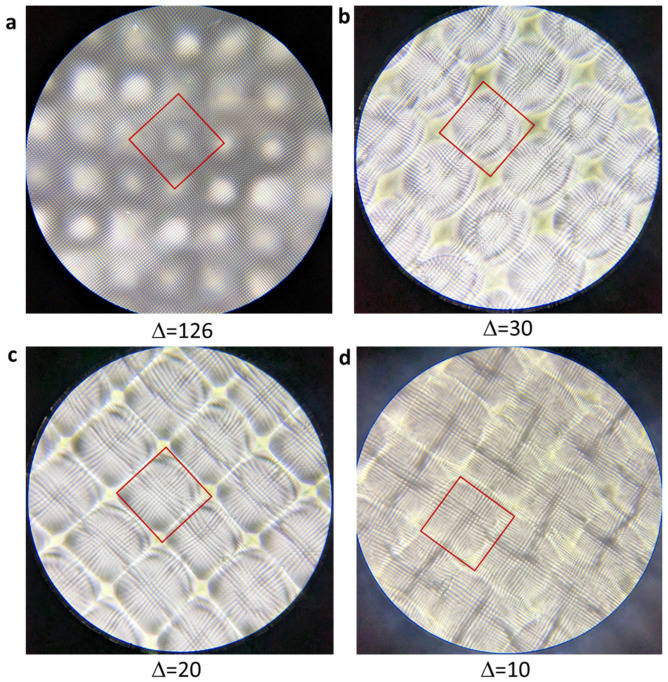
Optical microscopy images of holographic structures fabricated by SLM-based interference lithography with the red squares indicating a single unit super-cell, and gray levels of (254, 128) and (254-∆, 128-∆) in the phase pattern, where ∆ = 126 (**a**), 30 (**b**), 20 (**c**), and 10 (**d**) respectively.

The simulation and prediction are then tested by experimental results. Through interference lithography, we fabricate holographic structures in DPHPA when ∆ = 126, 30, 20, and 10 in the gray level of the phase pattern in Figure 1b. In our over-exposed sample in Figure 3, we can observe the change in dark region size. ∆ = 126, 30, 20, and 10 correspond to intensity ratios I_inner_/I_outer_ =0.1, 0.08, 0.04, and 0.03, respectively. As observed in Figure 3a–d, the dark region becomes smaller with decreasing intensity ratio, following the trend of simulation prediction. In Figure 3a, near 50% bright regions and 50% dark regions are observed. For ∆ = 30, 20, and 10, the unit super-cells are clearly defined by red squares in Figure 3b–d. The corners of red squares are for dark regions, becoming smaller from (b), (c) to (d). The boundary of the super-cell is barely visible in Figure 3d.

**Figure 4:**
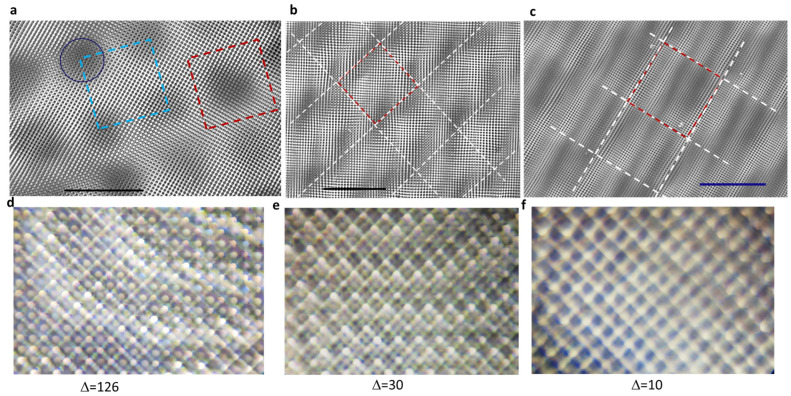
Scanning electron microscopy (SEM) images of the holographic structures fabricated by SLM-based interference lithography, with gray levels of (254, 128) and (254-∆, 128-∆) in the phase pattern, where ∆ = 126 (**a**), 30 (**b**), and 10 (**c**). Dashed blue and red squares indicate the unit super-cell. The dashed white lines in (**b**) connect dark regions and in (**c**) are for eye-guidance of similar features. Scale bars in solid dark lines represent 50 microns. (**d**–**f**) Optical images of Talbot diffraction patterns from the samples in (**a**–**c**), respectively.

Figure 4a shows a scanning electron microscopy (SEM) image of a holographic structure that is the same as that in Figure 3a. Unit super-cells are indicated by the dashed blue and red squares; the dark region is indicated by the circle; the red square in Figure 4b is the unit super-cell of the moiré photonic crystal; and dark regions are located at the crossing points of the dashed white lines in Figure 4b. Although the sample is not uniform due to the liquid DPHPA mixture, the appearance of an almost equal area of dark and bright regions, a square unit cell, and different motifs in dark and bright regions confirms the simulation results. Figure 4b,c shows well-developed holographic structures with ∆ = 30 and 10 in the gray levels (254, 128) and (254-∆, 128-∆) of the phase pattern. The appearance of curved surfaces might be due to the moving liquid mixture in the vertical mounting of the sample during exposure, particularly in the current proof-of-concept stage. To eliminate these curved surfaces, an alternative approach is to position the sample in a horizontal plane or employ a solid negative resist such as SU-8. It is difficult to locate the dark region in the well-developed holographic structure in Figure 4c; however, it is easier to draw the unit super-cell along the square lattice of the photonic crystal in the dashed red square in Figure 4c because of the predicted near uniform pattern in Figure 2b. Overall, SEM images deliver the expected holographic structure as predicted by the simulation.

We acknowledge that well-developed 3D structures can be achieved using SU-8 instead of DPHPA. Additionally, the presence of curved surfaces complicates the assessment of dark regions in 3D moiré photonic crystals. To determine the presence of these dark regions, Talbot diffractions were measured and captured in optical images, as depicted in Figure 4d–f for the corresponding samples shown in Figure 4a–c. Figure 4d displays patterns of squares and circles within the squares. In Figure 4e, square patterns and cross features within the squares are observed. Talbot diffraction patterns show that periodic bright dots appear above the dark regions when the microscope lens is close to the sample surface (Figure 4c). Furthermore, when the lens is moved away from the sample surface, a square pattern is formed, as depicted in Figure 4f. These observations provide evidence for the existence of dark regions.

**Figure 5:**
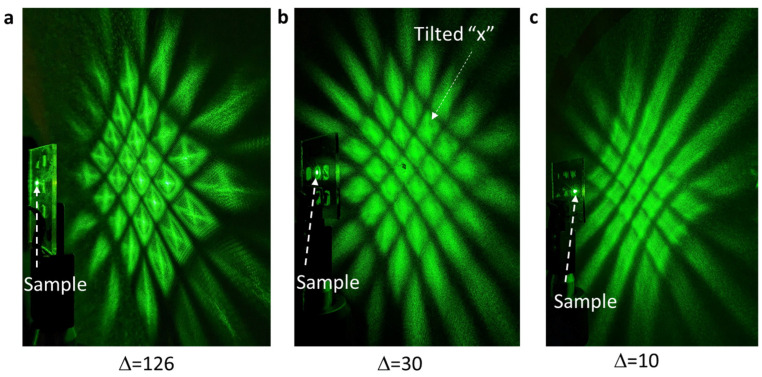
Diffraction patterns of the 532 nm laser from the holographic structures fabricated by SLM-based interference lithography, with gray levels of (254, 128) and (254-∆, 128-∆) in the phase pattern, where ∆ = 126 (**a**), 30 (**b**), and 10 (**c**). Sample locations are labeled.

The diffraction pattern has been used to judge whether the sample is overexposed or not [50], with a well-developed sample usually showing diffraction zones instead of diffraction spots in the 3D moiré photonic crystal [50]. Figure 5a–c shows the diffraction pattern of the 532 nm laser from the 3D moiré photonic crystals in Figure 4a–c, respectively. The diffraction pattern can be explained by the simulated pattern. Due to the large difference in the filling fraction of dielectric materials inside the red square and white square in Figure 2h, diffraction in the x- or y-directions is destroyed or becomes weak, while diffraction along the diagonal direction is strong due to the symmetric pattern along the dashed red lines in Figure 2d. These features result in a tilted “x” pattern in Figure 5a. Furthermore, the pattern along the dashed red line is uniform, square, symmetrical, and has the smallest feature that gives a diffraction outside and surrounding the “x” pattern, in agreement with the image in Figure 5a. On the other hand, for the almost uniform pattern in Figure 2a, square diffraction zones are expected, as in Figure 5c. For the pattern in Figure 2b,f, the mixture of diffraction patterns in Figure 5a,c is expected. The tilted “x” pattern is barely seen, as indicated by the white arrow in Figure 5b. Overall, SEM and the diffraction pattern from the holographic structures are explained well with the help of simulations. The knowledge gained provides guidance for future pixel-by-pixel phase engineering in SLM for complex holographic structures.

## 5. Discussion

**Figure 6:**
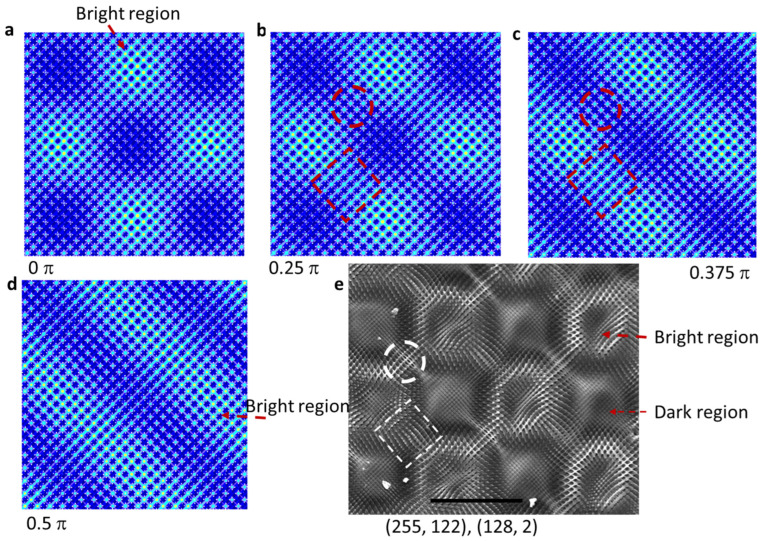
(**a**–**d**) Simulated x–y plane interference patterns of nine beams with a phase shift of (I_2_, I_4_) beams relative to the (I_1_, I_3_) beams of 0 π (**a**), 0.25 π (**b**), 0.375 π (**c**), and 0.5 π (**d**). (**e**) SEM image of the holographic structure in DPHPA fabricated by SLM-based lithography, with gray levels (255, 122) in quadrants (II, IV) and (128, 2) in quadrants (I, III). The scale bar in the SEM image represents 50 microns. The dashed red squares and circles in simulations in (**b**,**c**) correspond to the dashed white square and circle in SEM in (**e**).

We have only considered the intensity ratio of inner beams over outer beams in previous sections. With a high gray level difference ∆ = 126, the relative phase shift of inner beams needs to be considered. It is not clear whether the phase of the inner beam depends on both the gray level of pixels in each quadrant of the phase pattern in Figure 1b and the number of pixels in each quadrant. We start with the simulation of interference patterns using gray levels of (254, 128) in quadrants (II, IV) and (254-∆, 128-∆) in (I, III) (where ∆ = 126, I_inner_/I_outer_ = 0.1) and show them in Figure 6a–d for a phase shift of (I_2_, I_4_) beams relative to (I_1_, I_3_) beams of 0 π, 0.25 π, 0.375 π, and 0.5 π, respectively. The x–y plane interference pattern changes from square symmetry in Figure 6a to parallel lines for bright regions in Figure 6d. In Figure 6b,c, the link intensity between bright regions, as indicated by the dashed red square, is stronger than the one indicated by the dashed red circle, corresponding to the links indicated by the dashed white square and circle in the SEM image of the holographic structure in Figure 6e, respectively. It means the phase shift of (I_2_, I_4_) beams relative to (I_1_, I_3_) beams produced by gray levels (255, 122) in quadrants (II, IV) and (128, 2) in quadrants (I, III) is between 0.25π and 0.375π. If the phase shift depends on the gray level difference, we can estimate the phase shift by 0.25 × (255 − 128) × 2π/255 = 0.25π and 0.25 × (122 − 2) × 2π/255 = 0.24π [18,25,51]. Future research can focus on the dependence of the phase shift on the number of pixels in each quadrant of the engineered phase pattern.

In the future, the photonic behavior of 3D moiré photonic crystals can be investigated in various aspects, including the photonic bandgap, the photonic resonant cavity, and the topological photonic effect. With the availability of high computational power, it is possible to calculate the photonic bandgap when the interference pattern is converted into binary structures of silicon and air [54]. The presence of different filling fractions of dielectric materials in the superlattices along the z-direction of 3D moiré photonic crystals leads to shifts in photonic bandgaps and the formation of resonant cavities within the structure. Additionally, the study of topological photonic effects, specifically the investigation of topological interfaces [55] along the dashed red line in Figure 2g,h, is necessary due to the strong square patterns in diffraction surrounding the “x” patterns in Figure 5a.

## 6. Conclusions

In summary, we have studied the holographic fabrication of 3D moiré photonic crystals using nine-beam interference generated by the engineered phase pattern displayed in SLM. These nine beams consist of four inner beams, four outer beams, and one central beam. We have systematically studied the dependence of moiré photonic crystal on the intensity ratio of inner beams over outer beams through simulation, holographic fabrication, structural characterization, and diffraction. Superlattices modulated in the z-direction of 3D moiré photonic crystals have been discovered. Future research directions regarding the photonic behavior of 3D moiré photonic crystals have been discussed. This study not only leads towards the holographic fabrication of 3D complex structures but also the understanding of interfering beam phase and intensity controls through phase engineering in SLM-based lithography.

## Data Availability

Data will be available upon request.
